# Refractory facial cutaneous B-cell pseudolymphoma successfully treated with dupilumab

**DOI:** 10.1016/j.jdcr.2024.02.007

**Published:** 2024-02-18

**Authors:** Kalisa Lum, Ruth Sanchez, Cici Zhou, Sophia Sangar, Harry Dao, Ashley Elsensohn

**Affiliations:** a School of Medicine, Loma Linda University Health, Loma Linda, California; b School of Medicine, California University of Science and Medicine, Colton, California; c Department of Dermatology, Loma Linda University Health, Loma Linda, California; d Department of Pathology and Human Anatomy, Loma Linda University Health, Loma Linda, California

**Keywords:** African American, B-cell pseudolymphoma, dupilumab, therapy, Type V skin

## Introduction

Cutaneous pseudolymphoma refers to a group of benign reactive T- or B-cell lymphoproliferative processes that occur in response to identifiable stimuli or without an identifiable etiology. In this article, we report a case of facial cutaneous B-cell pseudolymphoma (CBPL) in Fitzpatrick type V skin that was unresponsive to treatment with high potency topical steroids, intralesional triamcinolone, hydroxychloroquine, and doxycycline but was successfully treated with dupilumab.^1^

## Case report

**Fig 1:**
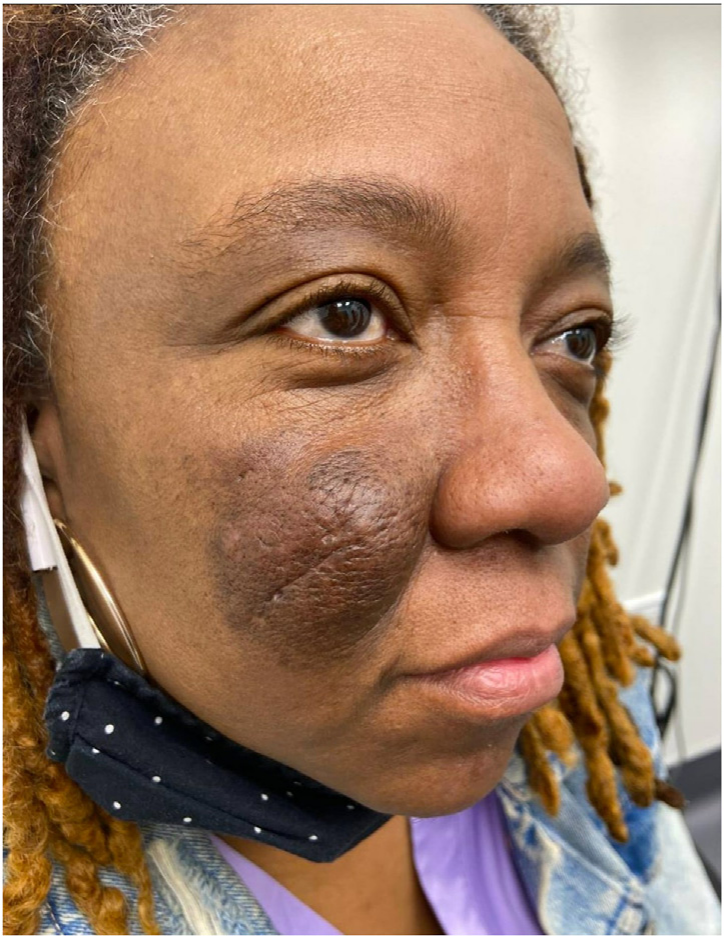
Right cheek before dupilumab treatment, demonstrating a firm nodule.

A 58-year-old African-American woman with Fitzpatrick skin type V presented with a 10-year history of a pruritic, hyperpigmented right cheek tumor (Fig 1), and a second but similar smaller tumor involving the left cheek. Both lesions began as patches that progressively enlarged. Community dermatologists had previously performed 5 biopsies of the right cheek with all of them demonstrating reactive lymphoid hyperplasia. Complete blood count with differential was unremarkable. Prior unsuccessful treatments included halobetasol and betamethasone, 5 mg/mL intralesional triamcinolone, and oral hydroxychloroquine.

**Fig 2:**
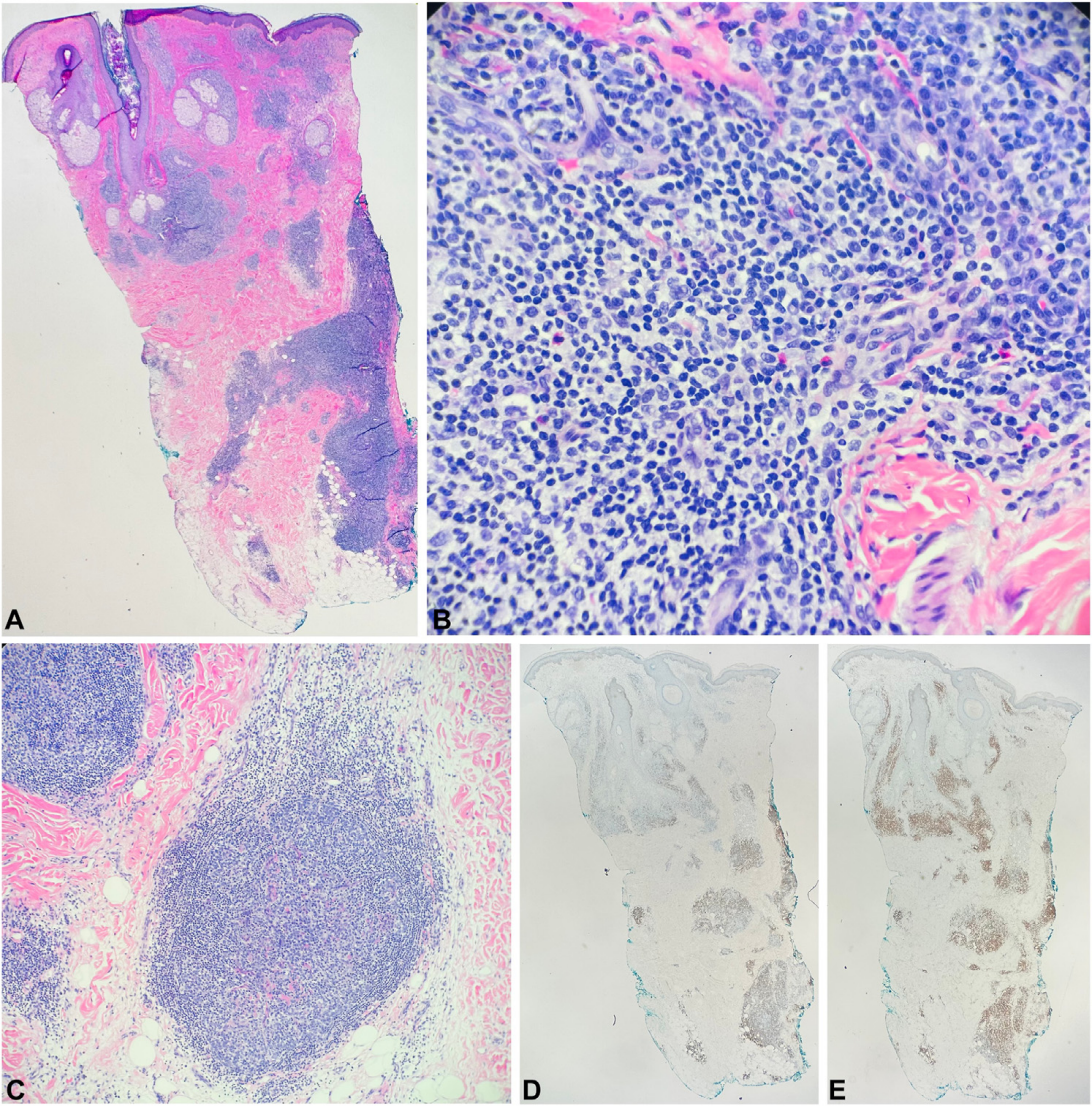
**A,** Superficial and deep dermal lymphoid infiltrate (hematoxylin and eosin, original magnification ×20). **B,** Lymphocytes and scattered eosinophils (hematoxylin and eosin, original magnification ×100). **C,** Follicular structure (hematoxylin and eosin, original magnification ×40). **D,** B-cells (immunohistochemistry for CD20, original magnification ×20, color enhanced). **E,** T-cells (immunohistochemistry for CD3, original magnification ×20, color enhanced).

An incisional biopsy performed at our institution revealed dense periadnexal and interstitial dermal lymphoid infiltrates with scattered eosinophils extending into the subcutis (Fig 2, *A* and *B*). Several lymphoid follicles with germinal cells of appropriate polarization and scattered tingible body macrophages were identified (Fig 2, *C*). Plasma cells and dermal fibrosis were not readily seen. Immunohistochemistry highlighted CD20, CD21, and BCL2 positive cells (Fig 2, *D*). The germinal centers were positive for BCL6 and focally for CD10. CD3 staining also highlighted several T-cells in the infiltrate (Fig 2, *E*). B-cell gene rearrangement was negative. Altogether, these findings were most compatible with B-cell pseudolymphoma.

**Fig 3:**
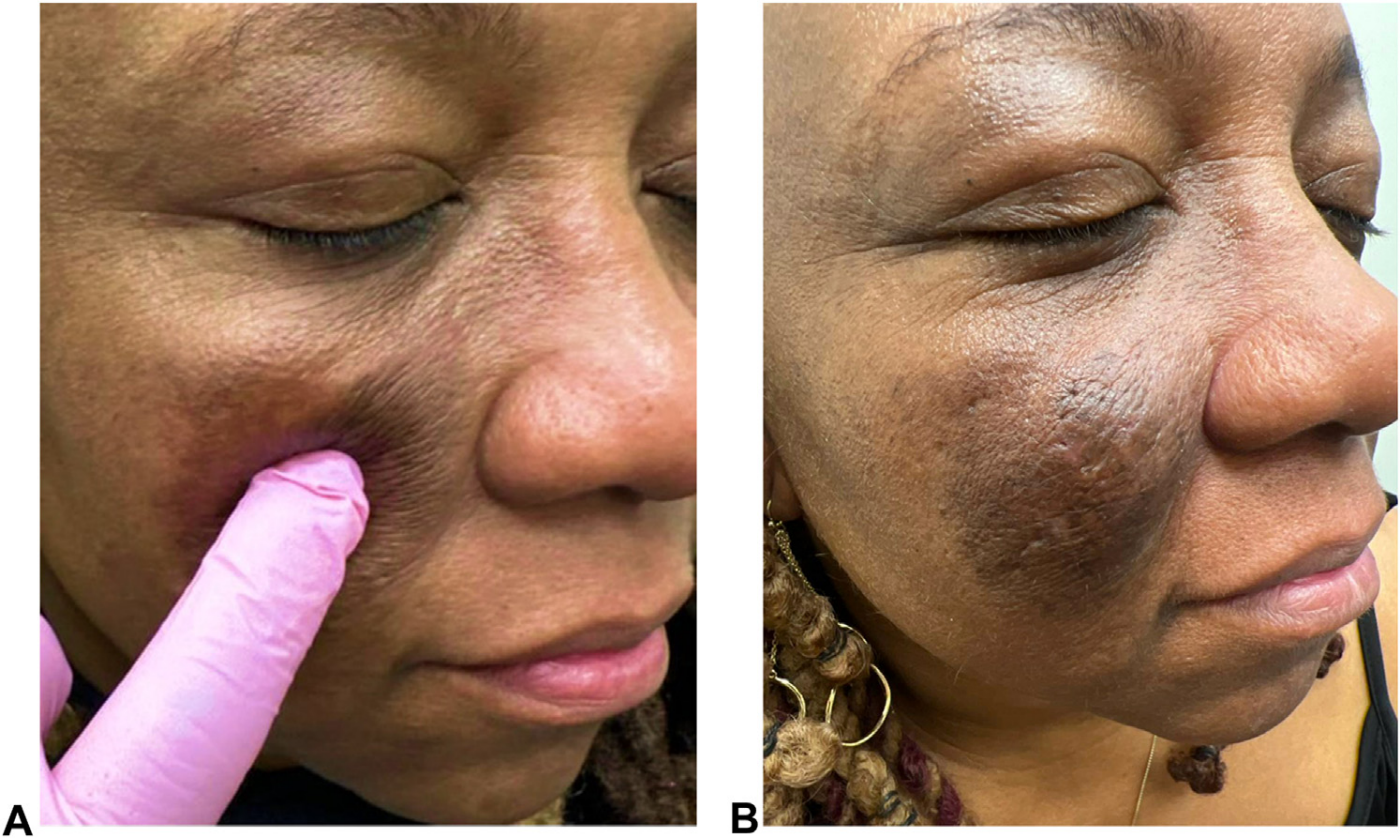
**A,** Right cheek after 9 months of dupilumab treatment, demonstrating easy compressibility of skin (color enhanced). **B,** Follow up after 9 months of dupilumab treatment exhibiting tumor resolution.

Intralesional triamcinolone was serially administered for pruritus, and doxycycline 100 mg twice daily was initiated. At 3-month follow up, there was no clinical improvement of either pruritus or lesion size. Subsequently, dupilumab was initiated with a 600 mg loading dose, followed by 300 mg every other week. Six months into treatment, there was significant improvement of both tumor size and pruritus without adverse effects. Nine months into dupilumab treatment, complete resolution of the tumors yielding easy compressibility of the involved sites was achieved, although postinflammatory hyperpigmentation and redundant skin from prior tissue expansion remained (Fig 3).

## Discussion

Causes of CBPL include insect bites, tattoos, vaccinations, trauma, and drugs, although CBPL is often idiopathic. Treatment for idiopathic CBPL varies, but generally involves topical and intralesional steroids. Refractory cases have improved with topical tacrolimus, hydroxychloroquine, interferon alpha, and thalidomide.^1^ The efficacy of dupilumab has been described in 2 cases with Fitzpatrick type II-III skin without residual post-inflammatory hyperpigmentation noted.^2^^,^^3^

Dupilumab is a monoclonal antibody that targets interleukin (IL)-4Rα, thereby blocking both IL-4 and IL-13.^4^ Food and Drug Administration-approved dermatologic indications include moderate-to-severe atopic dermatitis and prurigo nodularis.^4^ Activated T helper 2 cells produce several ILs, including IL-4 and IL-13, and promote the proliferation of inflammatory cells such as B-cells, mast cells, and eosinophils. By reducing T helper 2-cell differentiation, dupilumab inhibits B-cell proliferation and IgE production, as well as histamine-induced endothelial permeability.^4^ IL-4R complexes also activate STAT6, a transcription factor necessary for T helper 2 cell differentiation.^4^ STAT6 expression enhances the expression of Bcl-xL, an anti-apoptotic protein; therefore, dupilumab-mediated decrease in STAT6 may also reduce B cell proliferation by increasing B cell apoptosis.^5^

We present a case of refractory CBPL with primary cutaneous follicle center lymphoma (PCFCL)-like architecture responsive to dupilumab therapy, similarly to Joshi et al and Sugiura et al (Table I). In all 3 cases, histopathology demonstrated a dense dermal mononuclear infiltrate in a PCFCL-like architecture. Tissue eosinophils were notable in our case and in review of histologic images also appeared to have been present in the Joshi et al case. It is unclear whether eosinophils were also present in the Sugiura et al as there was no specific mention as to presence or absence of tissue eosinophils.

**Table I:** Patients treated with dupilumab for cutaneous B-cell pseudolymphoma

Source	Ethnicity	Immunostains	Molecular studies	Prior therapies	Dupilumab dosage, mg	Therapy, months	Outcome
Joshi et al^2^	White	CD20+ B-cells, CD3+ T-cells, BCL2 negative in germinal centers	Negative Ig heavy chain and T-cell receptor gene rearrangement	Topical steroids, doxycycline, hydroxychloroquine, methotrexate, rituximab	600, then 300 every 2 wk until resolution	12	No recurrence at 7-mo follow-up or 6 mo after therapy cessation
Sugiura et al^3^	Japanese	CD20+ B-cells, CD3+ T-cells, BCL2- and BCL6+ germinal centers	Negative Ig heavy chain and T-cell receptor gene rearrangement	Topical steroids, minocycline, doxycycline	600, then 300 every 2 wk until resolution	271∗	No recurrence at 5-mo follow-up
Lum et al.	African-American	CD20+ B-cells, CD3+ T-cells, BCL2- and BCL6+ germinal centers	Negative B-cell gene rearrangement	Topical steroids, triamcinolone, tacrolimus, hydroxychloroquine, doxycycline	600, then 300 every 2 wk until resolution	101∗	No recurrence at 9-mo follow-up

∗ Dupilumab therapy is ongoing.

Various treatments were trialed without significant resolution of symptoms in all 3 cases, leading to the initiation of dupilumab with a loading dose of 600 mg followed by 300 mg every 2 weeks (Table I). There has been no recurrence at initial follow-up, and no adverse effects have been reported with dupilumab therapy in any of the patients (Table I). Per Sugiura et al, the frequency of dupilumab was reduced to every 4 weeks at the 28th dose, and the patients in both our case and Joshi et al’s case have self-titrated the frequency of dosing to every 3 to 4 weeks once achieving clinical resolution.

The presence of certain histopathologic features, such as PCFCL-like architecture, infiltrate density, or possibly even tissue eosinophils, may correlate with therapeutic response. This case highlights an increasing number of cases that have successfully utilized dupilumab therapy for CBPL with PCFCL-like growth pattern. Future studies should explore the effectiveness of dupilumab for other B-cell pseudolymphoma subtypes, long-term remission rates, and clinical and histopathologic features associated with treatment response.

## Conflicts of interest

None disclosed.
